# 

**Published:** 1873-02

**Authors:** 


The Journal is published Monthly, at Three Dollars per annum, in advance. Each Number contains sixty-four Pages.

### ATLANTA MEDICAL & SURGICAL JOURNAL.

EDITED BY

JOSEPH P. LOGAN, M.D.,

AND

W. F. WESTMORELAND, M.D.,

*Professor of the Principles and Practice of Surgery in Atlanta Medical College.*

Vol. X.—FEBRUARY, 1873.—No. 11.

*PAX ET SCIENTIA, SED VERITAS SINE TIMORE.*

ATLANTA, GEORGIA:

HERALD PUBLISHING COMPANY'S PRESS.

1872.

